# Biocontrol of Bacterial Fruit Blotch by *Bacillus subtilis* 9407 via Surfactin-Mediated Antibacterial Activity and Colonization

**DOI:** 10.3389/fmicb.2017.01973

**Published:** 2017-10-11

**Authors:** Haiyan Fan, Zhanwei Zhang, Yan Li, Xun Zhang, Yongming Duan, Qi Wang

**Affiliations:** Department of Plant Pathology, College of Plant Protection, China Agricultural University, Beijing, China

**Keywords:** *Bacillus subtilis*, Acidovorax citrulli, surfactin, antibacterial activity, colonization, biological control

## Abstract

In this study, *Bacillus subtilis* 9407 showed a strong antibacterial activity against *Acidovorax citrulli in vitro* and 61.7% biocontrol efficacy on melon seedlings 4 days post inoculation under greenhouse conditions. To understand the biocontrol mechanism of *B. subtilis* 9407, identify the primary antibacterial compound and determine its role in controlling bacterial fruit blotch (BFB), a *srfAB* deletion mutant (Δ*srfAB*) was constructed. The Δ*srfAB* which was deficient in production of surfactin, not only showed almost no ability to inhibit growth of *A. citrulli* but also decreased biofilm formation and reduced swarming motility. Colonization assay demonstrated that *B. subtilis* 9407 could conlonize on melon roots and leaves in a large population, while Δ*srfAB* showed a four- to ten-fold reduction in colonization of melon roots and leaves. Furthermore, a biocontrol assay showed that Δ*srfAB* lost the biocontrol efficacy. In summary, our results indicated that surfactin, which consists of C13- to C16-surfactin A was the primary antibacterial compound of *B. subtilis* 9407, and it played a major role in biofilm formation, swarming motility, colonization and suppressing BFB. We propose that the biocontrol activity of *B. subtilis* 9407 is the results of the coordinated action of surfactin-mediated antibacterial activity and colonization. This study reveals for the first time that the use of a *B. subtilis* strain as a potential biological control agent could efficiently control BFB by producing surfactin.

## Introduction

Bacterial fruit blotch (BFB), caused by the Gram-negative bacterium *Acidovorax citrulli* (syn. *Acidovorax avenae* subsp. *citrulli*), is a serious disease threat to cucurbit crops worldwide (Schaad et al., 2008; Bahar et al., 2009). The pathogen *A. citrulli* is mainly seed borne and infects organs of cucurbit plant at all stages of growth, resulting in seedling lesions, blight or fruit rot (Hopkins and Thompson, 2002; Popovic and Ivanovic, 2015). Currently, there are few commercially reliable sources of disease resistance to BFB in the cucurbit cultivars, and chemical and physical measures have limited efficacy for disease management (Hopkins et al., 2003; Burdman and Walcott, 2012). Due to difficulties in controlling BFB, and the highly destructive potential of BFB, safe and effective strategies for the prevention and cure for BFB are needed.

Biological control using microbial antagonists has received a great deal of attention as an alternative and promising measure to control different plant diseases (Daguerre et al., 2014; Chowdhury et al., 2015). Many antagonistic microorganisms including *Bacillus* spp., *Trichoderma* spp., *Streptomyces* spp., *Pseudomonas* spp., *Candida* spp. have been exploited and investigated against different plant pathogens (Pérez et al., 2015; Wang et al., 2015). Some microorganisms have been screened and tested the activity against BFB such as *Bacillus* spp., *A. avenae, Pichia anomala, Streptomyces* spp. (Fessehaie and Walcott, 2005; Wang et al., 2009; Jiang et al., 2015). *Bacillus* genus is one of the most frequently studied biological control agents. However, only limited attempts have been made to control BFB using *Bacillus*. To date, there are no reports about using *B. subtilis* as a biological control agent against BFB.

Multiple modes of action of *Bacillus* spp. were reported to contribute to the biological control, including antibiosis, competition, and induce host systemic resistance (Chowdhury et al., 2015). The production of non-ribosomally synthesized antibiotics, especially surfactin, iturin, and fengycin, plays an important role in suppressing diseases (Zeriouh et al., 2011; Guo et al., 2014). The surfactin family is constituted of a seven amino-acid peptide ring linked to a β-hydroxy fatty acid consisting of 13–16 carbon atoms and synthesized by four biosynthetic genes *srfAA, srfAB, srfAC* and *srfAD* (Falardeau et al., 2013). It has been reported that surfactin displays significant inhibitory activity against pathogenic fungi, bacteria, viruses and mycoplasmas (Vollenbroich et al., 1997; Hwang et al., 2008; Wen et al., 2011; Sabate and Audisio, 2013).

Successful colonization of biological control agents on the plants is essential for biocontrol efficacy (Chowdhury et al., 2013; Weng et al., 2013). The ability of *Bacillus* strains to colonize on plants depends on various factors including swarming motility and biofilm formation (Yaryura et al., 2008; Gao et al., 2016). Swarming motility is a social form of motility that provides flagellated bacteria with the ability to travel rapidly to a nutrient-rich environment and colonize within advantageous colonization locations. Biofilms are multicellular communities of differentiated cells encased by an extracellular matrix, which provide protection against environmental insults and facilitate interactions with other organisms (Kolter and Greenberg, 2006). Studies have revealed that surfactin also plays important roles in biofilm formation, motility, colonization on host plant tissues, and induce plant resistance against pathogens (Ghelardi et al., 2012; Zeriouh et al., 2014; Luo et al., 2015; Rahman et al., 2015). For example, *B. subtilis* UMAF6614 produces surfactin to ensure well colonize on melon phylloplane and contribute to the biocontrol activity (Zeriouh et al., 2014). Aleti et al. (2016) reported that the surfactin variants with subtle structural differences have varying signal strengths on biofilm formation and root colonization and act specifically on the respective producing strain. However, the role of surfactin in the biocontrol of BFB has not yet been reported.

*Bacillus subtilis* 9407 was isolated from healthy apples from an infested orchard. In the previous study, we showed that fengycin is the primary antifungal compound of *B. subtilis* 9407, and it plays a major role in suppressing apple ring rot disease (Fan et al., 2017). In this study, we showed that *B. subtilis* 9407 effectively suppressed *A. citrulli in vitro* and *in vivo*. However, the primary antimicrobial compound involved in its antibacterial activity and the role of the primary antimicrobial compound in biocontrol are not understood. Lipopeptide crude extracts from *B. subtilis* 9407 showed strong antibacterial activity against *A. citrulli* MH21. Therefore, we mutated candidate genes for biosynthesis of selected lipopeptide and investigated the antibacterial activity of the mutants against *A. citrulli* MH21. Since Δ*srfAB* almost completely lost the ability to inhibit the growth of *A. citrulli* MH21, we focused on surfactin, indicated it was a mixture of C13- to C16-surfactin A. Then, we investigated the putative contribution of surfactin to the biofilm formation, swarming motility, colonization, and biocontrol abilities of this biocontrol agent. We demonstrated that surfactin was the primary antibacterial compound of *B. subtilis* 9407, and it played a major role in biofilm formation, swarming motility, colonization and suppressing BFB. We propose that the biocontrol activity of this strain is the results of the coordinated action of antibacterial activity and colonization. The results of our study may provide a new biological control agent for controlling BFB and improve our understanding of the biocontrol mechanism of *B. subtilis* 9407.

## Materials and Methods

### Bacterial Strains, Plasmids and Growth Conditions

The bacterial strains and plasmids used in this study are described in Supplementary Table S1. *B. subtilis* strains were routinely grown at 37°C in Luria-Bertani (LB) medium. For assays of biofilm formation, MSgg medium was used. The recipe for MSgg is as follows: 5 mM potassium phosphate (pH 7.0), 100 mM MOPS (morpholine propane sulfonic acid) (pH 7.0), 2 mM MgCl_2_, 700 μM CaCl_2_, 50 μM MnCl_2_, 50 μM FeCl_3_, 1 μM ZnCl_2_, 2 μM thiamine, 0.5% glycerol, 0.5% glutamic acid, 50 μg/mL tryptophan, 50 μg/mL threonine, and 50 μg/mL phenylalanine (Branda et al., 2001). *Escherichia coli* DH5α was used as a host for molecular cloning and grown at 37°C in LB medium. *A. citrulli* MH21 was grown at 28°C in LB medium (Ren et al., 2014). When required, antibiotics were added at the following concentrations for growth of *B. subtilis*: 5 μg/mL of chloramphenicol, 10 μg/mL of tetracycline. For growth of *E. coli* and *A. citrulli* MH21, antibiotics were added at the following concentrations: 100 μg/mL of ampicillin and 50 μg/mL of ampicillin, respectively.

### DNA Manipulations

The general methods for molecular cloning were performed according to standard procedures (Sambrook and Russell, 2001). Restriction enzymes and other enzymes for molecular cloning were all purchased from Takara Co., Ltd. (Dalian, China). PCR products were purified with a PCR purification kit (Tiangen Biotech Co., Ltd., Beijing, China) and used according to the manufacturer’s instructions. The plasmids were isolated using a plasmid mini-prep kit purchased from Bioteke Bio Solutions Co., Ltd. (Beijing, China). *E. coli* DH5α cells were transformed by heat shock transformation. *B. subtilis* cells were transformed by electroporation using a Gene-Pulser (Bio-Rad, MicroPulser, United States), as recommended by the manufacturer. Oligonucleotide primer synthesis and DNA sequencing services were performed at Tsingke Biological Technology Co., Ltd. (Beijing, China).

### Antibacterial Activity Assay and Minimum Inhibitory Concentration Determination

The antagonistic activity of *B. subtilis* 9407 against *A. citrulli* MH21 was roughly analyzed as previously described (Wu et al., 2015), with some modifications. Fresh *A. citrulli* MH21 plates were prepared for the assay. When the concentration of *A. citrulli* MH21 grown in LB medium at 28°C was up to 4 × 10^7^ CFU/mL, 5 mL bacteria suspension was mixed with 300 mL melting LB agar and cooled below 60°C to prepare the plates. The 1 μL of an overnight culture of *B. subtilis* 9407 was spotted onto the surface of the plate which was then incubated at 28°C for 48 h to observe the growth inhibition effect. The diameters of inhibition zones were then measured and recorded. The other plant pathogens tested in this study were supplied by the Department of Plant Pathology in the College of Plant Protection at China Agricultural University, China.

To analyze the antibacterial activity of the extraction of lipopeptides from *B. subtilis* 9407, 10 μL of the appropriate dilutions of lipopeptide crude extracts were loaded into wells punched in *A. citrulli* MH21 plates prepared by the method described above. The plates were incubated at 28°C for 48 h and the antibacterial abilities of the lipopeptide crude extracts were assessed by observing the resulting inhibition zones.

The inhibiting activity of Δ*srfAB* and Δ*ppsB* against *A. citrulli* MH21 was also tested by spotting bacterium on *A. citrulli* MH21 plates prepared by the method described above. *B. subtilis* 9407 used as a control was also spotted. Then the plates were incubated at 28°C for 48 h to observe the growth inhibition effect.

The inhibiting activity of the extraction of lipopeptides from wild-type strain 9407, Δ*srfAB* and Δ*ppsB* were also tested by spotting 10 μL of lipopeptide crude extracts into wells punched in *A. citrulli* MH21 plates according to the method described above.

The minimum inhibitory concentration (MIC) of lipopeptide crude extracts from *B. subtilis* 9407 against *A. citrulli* MH21 was determined as previously described (Bais et al., 2004). In brief, *A. citrulli* MH21 was first grown in 5 mL LB broth to 4 × 10^5^ CFU/mL, and 190 μL of culture was added to 96-well microtiter plates. Then, 10 mL of serial 2-fold dilutions of lipopeptide crude extracts was mixed with culture in 96-well plates. Ten microliters of the methanol was used as control. The MIC was visually defined as the lowest concentration of an antibiotic that completely inhibited cell growth after incubation for 24 h at 28°C. All susceptibility trials were conducted in triplicate.

The MIC of surfactin standard for *A. citrulli* MH21 was determined according to the method described above. Four microliters of the serial 2-fold dilutions of commercial surfactin was mixed with 196 mL of *A. citrulli* MH21 culture in 96-well plates. Commercial surfactin (Sigma–Aldrich, St. Louis, MO, United States) was dissolved in dimethyl sulfoxide (DMSO). Four microliters of the DMSO was used as control.

### Construction of *srfAB* Marked Deletion Mutant in *B. subtilis* 9407

The *srfAB* marked deletion mutant was constructed using the temperature-sensitive suicide plasmid pMAD as described previously (Arnaud et al., 2004). A tetracycline resistance gene (Tet) was amplified by polymerase chain reaction (PCR) from plasmid pGFP78 (Gao et al., 2015) using primer pair Tet-F and Tet-R (All primer sequences are shown in Supplementary Table S2). The resulting fragment was digested with *Spe*I/*Pst*I and cloned into pEBS (Wang et al., 2007), which had also been digested with *Spe*I/*Pst*I, generating pEBST. Regions that were upstream and downstream of the *srfAB* gene were amplified from *B. subtilis* 9407 genomic DNA using the primer pairs srfAB-Up-F/srfAB-Up-R and srfAB-Dn-F/srfAB-Dn-R, respectively. These fragments were digested with *Sal*I/*Pst*I and *Spe*I/*Sac*I, respectively, and were cloned into the pEBST plasmid to create pEBST-srfAB. The Up-Tet-Dn fragment was amplified from pEBST-srfAB using the primer pair srfAB-F and srfAB-R. The resulting fragment was digested with *Bgl*II/*Mlu*I and cloned into pMAD, which had also been digested with *Bgl*II/*Mlu*I, generating pMAD-srfAB. The pMAD-srfAB plasmid was subsequently mobilized into *B. subtilis* 9407 by electroporation. Transformants were obtained after incubation at 30°C for 2 days on LB plates containing erythromycin and X-Gal (40 μg/mL). The allelic replacement of pMAD-srfAB in *B. subtilis* 9407 was performed according to the published protocol (Arnaud et al., 2004). Erythromycin-sensitive clones were isolated, and the mutants were identified by PCR with primer pair srfAB-F and srfAB-R and subsequently confirmed by Sanger sequencing.

### Extraction of Lipopeptides

Lipopeptide extraction was performed as previously described (Chen et al., 2016). In brief, after cultivating the cells in 50 mL Landy medium at 30°C for 72 h, the cell-free supernatant was obtained by centrifugation at 6,000 × *g* for 10 min and filtration using a bacterial filter (φ = 0.22 μm). The column (Sigma Amberlite) containing 6 g of XAD16 adsorbent resin (Sigma–Aldrich, St. Louis, MO, United States) was washed with 50 mL of deionized water to remove salts. The cell-free supernatant was passed through the XAD16 resin column, washed with deionized water and eluted with 14 mL of 100% methanol. The lipopeptide crude extracts were dissolved in 1 mL of methanol, followed by drying with a rotary evaporator.

### Semipreparative Reverse-Phase High Performance Liquid Chromatography (RP-HPLC)

The samples were run on an RP-HPLC equipped with Waters Sunfire C18 columns (5 μm, 4.6 × 150 mm) at room temperature, with a flow rate of 1.0 mL/min. The mobile phase consisted of 0.1% trifluoroacetic acid (TFA) in HPLC grade water (solvent A) and 0.1% TFA in HPLC grade acetonitrile (solvent B). The elution was monitored by determining the absorbance at 214 nm. Commercial surfactin (Sigma–Aldrich, St. Louis, MO, United States) was used as standards.

### Liquid Chromatography Electrospray Ionization Tandem Mass Spectrometry (LC-MS/MS) Analysis

Quattro Premier XE tandem quadrupole mass spectrometer (Waters) was used for LC-MS/MS analysis. The Waters Sunfire C18 column (4.6 × 150 mm, 5 μm) was used for liquid chromatography at 1.0 mL/min flow rate, at 25°C. The mobile phase consisted of solvent A and solvent B. Solvent A was water, eluent B was acetonitrile (ACN), both containing 0.1% formic acid (FA). The injection volume was 10 μL of surfactin standard (1 mg/mL) or lipopeptide crude extracts from *B. subtilis* 9407. The elution was monitored by determining the absorbance at 214 nm. The mass spectral was analyzed in both the negative ion and positive ion mode (ESI+/ESI-), the other parameters were as follows: 120°C source temperature; voltages were 3.2 kV for the capillary and 30 V for the cone voltage, 350°C desolvation temperature. Surfactin standard was purchased from Surfactin (Sigma–Aldrich, St. Louis, MO, United States).

### Analysis of Biofilm Formation

The biofilm formation was analyzed in MSgg medium by using the method described previously (Yan et al., 2016). The wild-type and Δ*srfAB* strains were first grown in 5 mL LB broth to late exponential growth phase (OD_600_ = 1.0), and 4 μL of culture was added to 4 mL of MSgg medium (a 1000-fold dilution) in 12-well St. microtiter plates and incubated statically at 25°C for 72 h.

In the experiments for the restoration of biofilm formation, 2 μL of the appropriate dilutions of commercial surfactin was mixed with 4 mL of MSgg medium in 12-well plates prior to inoculation with Δ*srfAB*. Commercial surfactin (Sigma–Aldrich, St. Louis, MO, United States) was dissolved in DMSO.

### Assays of Swarming Motility

Swarming motility assays of wild-type *B. subtilis* 9407 and Δ*srfAB* were performed according to Chen et al. (2012). In brief, 5 mL LB liquid cultures were prepared with shaking (200 rpm) at 37°C to mid-log phase, 1 mL of cells were collected by centrifugation at 6,000 × *g* for 5 min, washed with phosphate-buffered saline (PBS; 137 mM NaCl, 2.7 mM KCl, 10 mM Na_2_HPO_4_, and 2 mM KH_2_PO_4_), and resuspended in 100 μL PBS. The swarming agar plates (LB solidified with 0.7% agar) were dried for 20 min in a laminar flow hood, centrally inoculated with 3 μL of the cell suspension, dried for another 10 min and incubated at 30°C for 6.5–7 h (the surface of the plates inoculated with wild-type cells had almost been fully covered by the swarming cells). Afterward, the swarming plates were removed to a laminar flow hood, dried for 1 h and incubated at room temperature for another 12 h to allow cell growth in order to clearly visualize the swarming zone. The diameter of the swarming zone was measured.

For extracellular complementation experiments, 2 μL of the appropriate dilutions of commercial surfactin (dissolved in DMSO) was spotted at the center of swarming agar plates and dried in a laminar flow hood before the motility assay.

### Colonization Assay

*B. subtilis* 9407 and Δ*srfAB* strains were marked with resistance gene using the shuttle vector pC-1 containing a chloramphenicol resistance gene. The plasmid pC-1 was transformed into *B. subtilis* 9407 and Δ*srfAB* by electroporation. Transformants were selected with chloramphenicol and then identified by PCR with primer pair pC-1-F and pC-1-R.

The melon (*Cucumis melo*) was used for colonization assays. Melon seeds were soaked in the 55°C water for 30 min, placed between the wet gauze, and incubated at 28°C for 36 h to germinate. The wild-type and Δ*srfAB* strains were grown in nutrient broth (NB) in a shaker at 30°C with 160 rpm for 48 h. The cells were harvested by centrifugation at 6,000 × *g* for 10 min and adjusted with PBS buffer (pH 7.0) to obtain the desired bacterial concentration (10^7^ CFU/mL). The germinated melon seeds were soaked in the bacterial suspension at room temperature for 30 min with gentle agitation. Seeds soaked in PBS buffer alone were used as the controls. After that the treated seeds were air-dried and sown in 600 mL black plastic pots (six seeds per pot) filled with sterile soil and vermiculite in a ratio of 2:1. Three pots were used for each replicate and three replicates were used for each bacterial strain. The pots were placed in a growth chamber at 28°C with a 16 h photoperiod and 60% humidity. Samples were collected at 0, 5, 10, 15, and 20 days post inoculation. To assays of cell colonization on roots and leaves, 0.1 g fresh weight of the melon roots and leaves were collected. Samples were surface sterilized by 3 min in sodium hypochloride (10% active chlorine) and washed three times with sterile water for at least 5 min each. The surface-sterilized samples were then disrupted in a sterile mortar and pestle. The suspensions were diluted by 10-fold serial dilutions in sterile water, and 100 μL of each diluted suspension was plated on LB agar plates supplemented with chloramphenicol (5 μg/mL) and then incubated at 37°C for 12 h. Bacterial colony-forming units on each plate were counted. The experiments were repeated three times.

### Biocontrol of Bacterial Fruit Blotch under Greenhouse Conditions

Melon was used to evaluate the biocontrol activity of wild-type strain 9407 and Δ*srfAB* against *A. citrulli* MH21. Melon seeds were pre-germinated as described above. The bacterial suspensions of *B. subtilis* 9407 and Δ*srfAB* were prepared by the method described in Section “Colonization Assay.” The germinated seeds were soaked in the bacterial suspension at room temperature for 30 min with gentle agitation and then air-dried. Seeds soaked in PBS buffer alone were used as the controls. Eight treated seeds were sown in 600 mL black plastic pots filled with soil and vermiculite in a ratio of 2:1. The pots were placed in a greenhouse with the following conditions: 28–30°C, 60% humidity, and 16 h of light alternating with 8 h of darkness. *A. citrulli* MH21 was cultured at 28°C for 48 h in LB broth. The cells were harvested by centrifugation at 5,000 × *g* for 15 min and the bacterial suspension was adjusted with PBS to 10^8^ CFU/mL. After sown for 3 days, both sides of the leaves were sprayed with bacterial suspension of *A. citrulli* MH21.

The seedlings were evaluated for BFB severity daily based on the disease index, as described previously (Bahar et al., 2009). The disease index of each leaf was rated using a scale of 0–6, where 0, no symptom; 1, 10% or less necrotic lesions on the leaves; 2–5, 11–25%; 26–50%; 51–75% and 76–90% necrotic lesions on the leaves, respectively; and 6, >90% necrosis of the leaves. The disease severity and biocontrol efficacy were calculated as follows:

Disease severity (%) = Σ the number of diseased leaves in each grade × grade/(total number of leaves investigated × the highest disease index) × 100.

Biocontrol efficacy (%) = (incidence rate in the control - incidence rate in the *Bacillus*-treated group)/ incidence rate in the control × 100.

Three pots were used for each replicate, and the values were recorded as the means of three replicates for each treatment. The experiments were repeated three times.

### Statistical Analysis

The data were statistically analyzed using SPSS software 20.0. Student’s *t*-tests were used to determine the significant differences.

## Results

### *B. subtilis* 9407 Showed Strong Antibacterial Activity against *A. citrulli*

**FIGURE 1:**
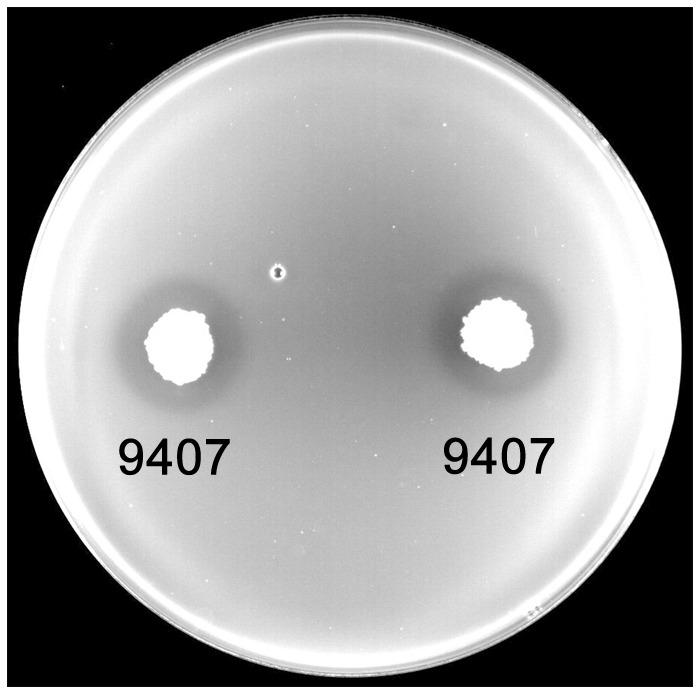
The antagonistic activity of *B. subtilis* 9407 against *A. citrulli* in a dual culture test on a LB plate.

To investigate the antibacterial activity of *B. subtilis* 9407 against *A. citrulli* MH21, a dual culture assay was conducted. Two days after inoculation, the inhibition zone of *B. subtilis* 9407 was 18.1 mm, suggesting that *B. subtilis* 9407 could observably inhibit the growth of *A. citrulli* MH21 (**Figure 1**). Moreover, *B. subtilis* 9407 showed significant antagonistic activity *in vitro* toward other pathogens that cause plant diseases in tomato, oilseed rape, Chinese cabbage, potato and other plants (**Table 1**).

**Table 1:** Antibacterial activity of *B. subtilis* 9407 determined in agar diffusion test.

Indicator strains	Diameter of the inhibition zone (mm)
*Pseudomonas syringae* pv. *tomato* DC3000	18.0
*Xanthomonas campestris* pv. *campestris* Xcc 8004	10.2
*Pectobacterium carotovora* subsp. *carotovora* Ecc 09	10.8
*Clavibacter michiganensis* subsp. *michiganensis* BT0505	0
*Pectobacterium atrosepticum* SCRI1043	15.8
*Acidovorax citrulli* MH21	18.3

### *B. subtilis* 9407 Exhibited High Biocontrol Efficacy on Bacterial Fruit Blotch

Next, we evaluated the biocontrol ability of *B. subtilis* 9407 against BFB caused by *A. citrulli* MH21 on melon under greenhouse conditions. We performed biocontrol assays using the *B. subtilis* 9407 strain pretreated seeds and inoculated *A. citrulli* MH21 3 days after planted (see “Materials and Methods”). Four days after *A. citrulli* MH21 inoculation, the disease severity of melon seedlings that were pre-treated with PBS (control) was 43.52% (**Table 2**). The disease severity of melon seedlings that were pre-treated with *B. subtilis* 9407 was 16.67%, for a noticeable reduction compared with the PBS treated control. Accordingly, the efficacy of *B. subtilis* 9407 in controlling the BFB caused by *A. citrulli* MH21 up reached 61.7%. Seven days post inoculation, the biocontrol efficacy of *B. subtilis* 9407 against BFB was 57.8% (**Table 2**). These results indicated that *B. subtilis* 9407 is a potential biological control agent for efficiently controlling BFB.

**Table 2:** Biocontrol assay of *B. subtilis* 9407 against bacteria fruit blotch under greenhouse condition.

Treatment	4 days post inoculation		7 days post inoculation	
	Disease	Biocontrol	Disease	Biocontrol
	severity (%)	efficacy (%)	severity (%)	efficacy (%)
*B. subtilis* 9407	16.67 ± 2.38^a^	61.7	27.78 ± 2.78^a^	57.8
Control	43.52 ± 5.26^b^	–	65.87 ± 8.36^b^	–

*The germinated melon seeds were soaked in bacterial suspensions of *B. subtilis* 9407 at 10^*7*^ CFU/ mL for 30 min. Phosphate-buffered saline (PBS) was used as control. After sown for 3 days, both sides of the melon leaves were sprayed with bacterial suspension of *A. citrulli* MH21(10^*8*^ CFU/mL). Three pots (eight seeds per pot) were used for each replicate. Data are presented as means of three replicates ± SD, and error bars represent SD for three replicates. Means with different letters have significant differences (*P* < 0.01).*

### Lipopeptide Crude Extracts from *B. subtilis* 9407 Showed Strong Antibacterial Activity against *A. citrulli* MH21

**FIGURE 2:**
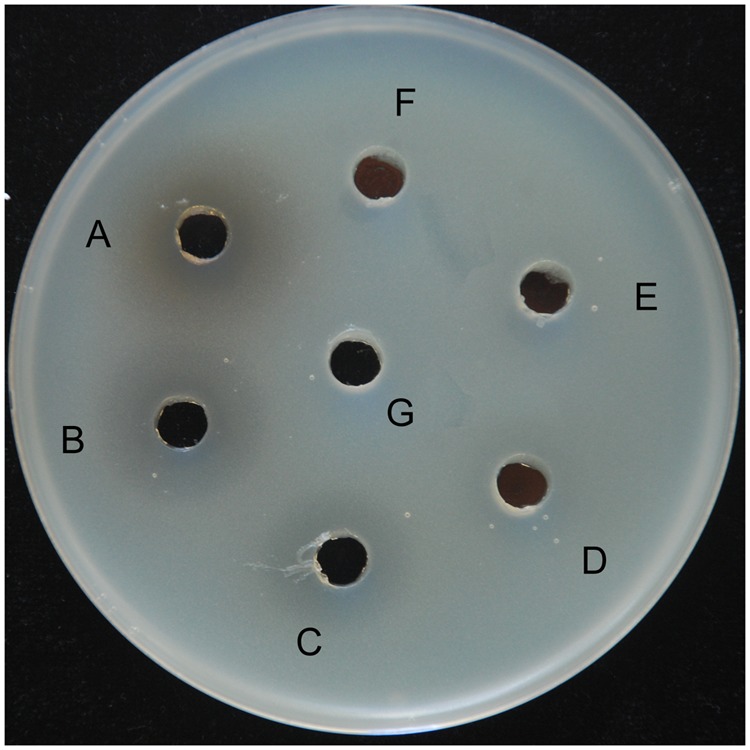
An antagonistic assay against *A. citrulli in vitro* by lipopeptide crude extracts from *B. subtilis* 9407. **(A)** lipopeptide crude extracts, **(B)** 2-fold dilution of lipopeptide crude extracts, **(C)** 5-fold dilution of lipopeptide crude extracts, **(D)** 10-fold dilution of lipopeptide crude extracts, **(E)** 50-fold dilution of lipopeptide crude extracts, **(F)** 100-fold dilution of lipopeptide crude extracts and **(G)** methanol.

To identify the primary antibacterial compound of *B. subtilis* 9407 against *A. citrulli*, the antibacterial activity of lipopeptide crude extracts from *B. subtilis* 9407 was tested against *A. citrulli* MH21 as described in Section “Materials and Methods.” Lipopeptide crude extracts from *B. subtilis* 9407 showed antibacterial activity against *A. citrulli* MH21, at the dilution rate from 0- to 10-fold (**Figure 2**). The MIC of lipopeptide crude extracts from *B. subtilis* 9407 for *A. citrulli* MH21 was determined to be 40 times dilution of lipopeptide crude extracts. These results indicated that lipopeptide crude extracts from *B. subtilis* 9407 showed strong antibacterial activity against *A. citrulli* MH21.

### The Δ*srfAB* Mutant Showed No Ability to Inhibit Growth of *A. citrulli* MH21

**FIGURE 3:**
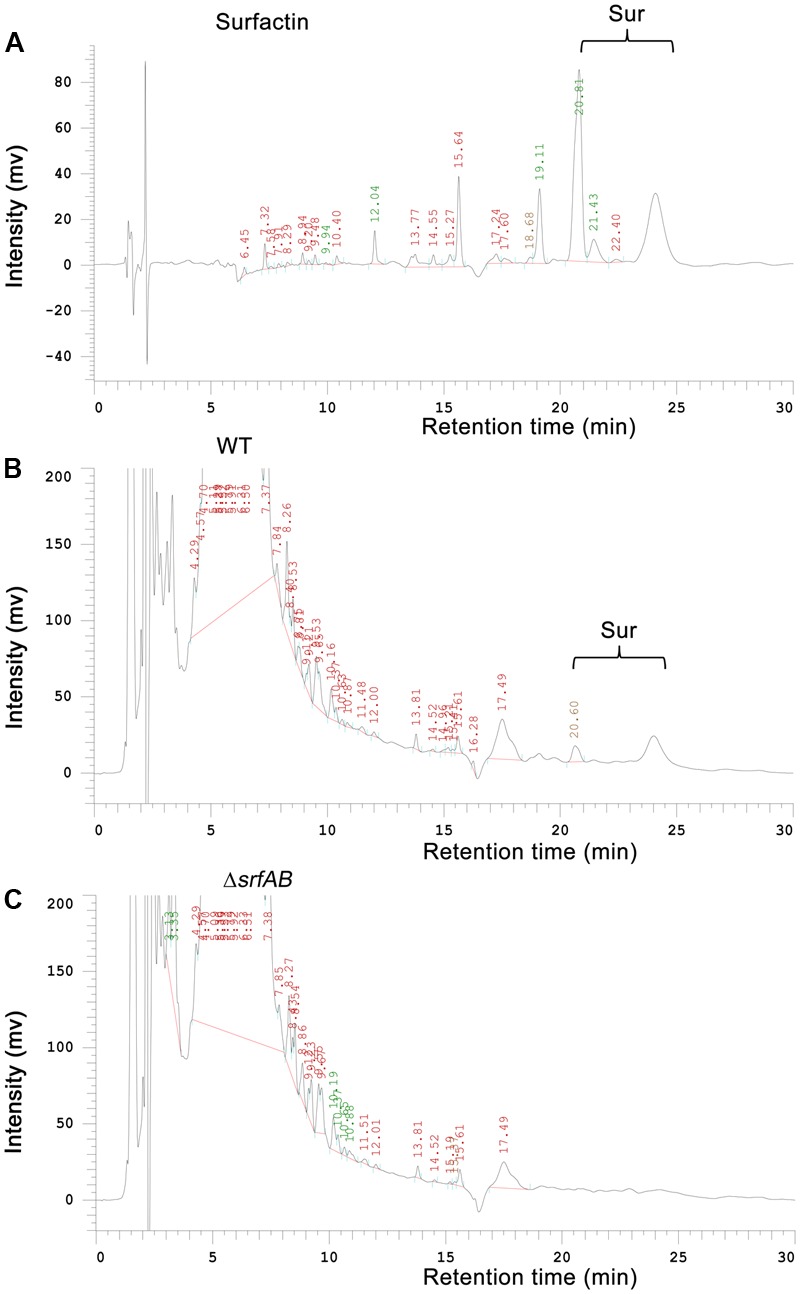
Semipreparative reverse-phase HPLC (RP-HPLC) analysis of lipopeptide crude extracts from wild-type *B. subtilis* 9407 and Δ*srfAB*. **(A)** Surfactin (Sigma–Aldrich, St. Louis, MO, United States), **(B)** wild-type *B. subtilis* 9407(WT) and **(C)** Δ*srfAB*.

**FIGURE 4:**
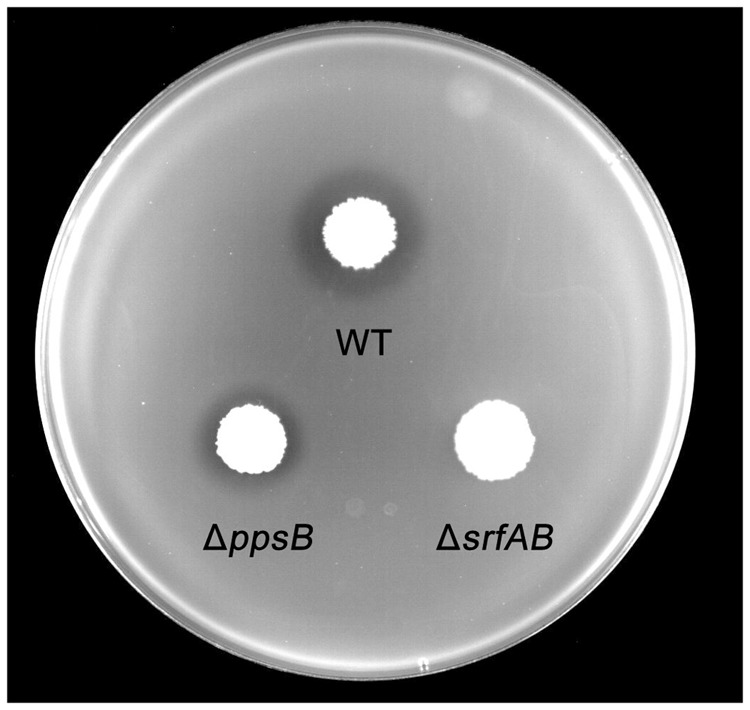
Antibacterial activity assay of *B. subtilis* 9407(WT), Δ*ppsB* and Δ*srfAB* against *A. citrulli* on a LB plate.

**FIGURE 5:**
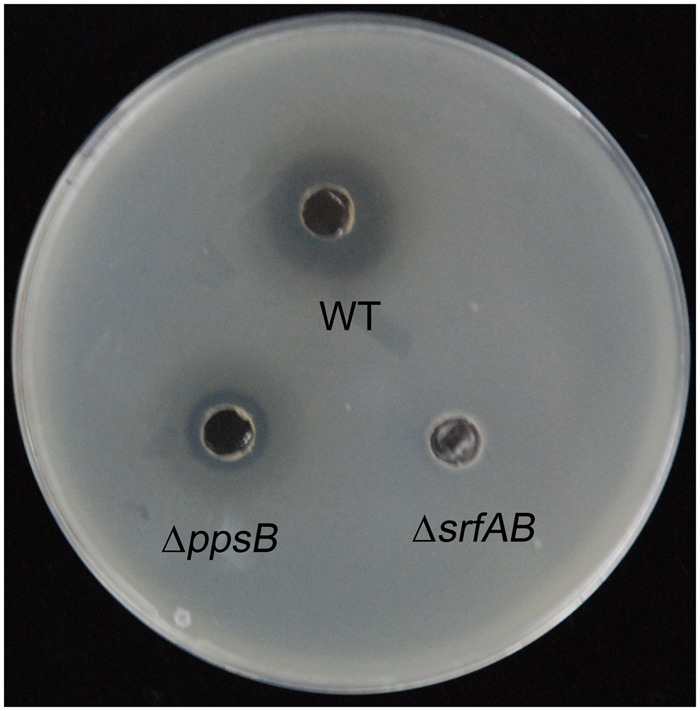
An antagonistic assay against *A. citrulli in vitro* by lipopeptide crude extracts from *B. subtilis* 9407(WT), Δ*ppsB* and Δ*srfAB*.

To further identify the primary antibacterial compound of *B. subtilis* 9407 against *A. citrulli*, we decided to mutate candidate genes for biosynthesis of selected lipopeptide, which were reported to show antibacterial activity. The selected candidate genes were *srfAB* and *ppsB*, responsible for the synthesis of surfactin and fengycin, respectively (Zeriouh et al., 2014; Fan et al., 2017). To generate a *srfAB* marked deletion mutant, the temperature-sensitive suicide plasmid pMAD-srfAB was constructed (see “Materials and Methods”) to disrupt the open reading frame of the *srfAB* gene, abrogating the production of surfactin. The surfactin synthesis abilities of the wild-type *B. subtilis* 9407 and Δ*srfAB* were further detected by RP-HPLC (**Figure 3**). It was found that wild-type *B. subtilis* 9407 produced surfactin, while Δ*srfAB* completely lost the ability to produce surfactin. In our previous work, we confirmed that the Δ*ppsB* was phenotypically characterized as non-fengycin producer (Fan et al., 2017). We tested the antibacterial activity of the mutants against *A. citrulli* MH21. Compared with the wild-type *B. subtilis* 9407, Δ*ppsB* showed a reduced antibacterial activity against *A. citrulli* MH21. However, Δ*srfAB* showed no clear zone of inhibition of the growth of *A. citrulli* MH21 (**Figure 4**). After that, lipopeptide crude extracts were subjected to analyze the antibacterial activity *in vitro*. Consistent with our hypothesis, lipopeptide crude extracts from Δ*srfAB* showed almost no inhibition of *A. citrulli* MH21 (**Figure 5**). These results suggested that surfactin is the primary active compound to exert the inhibitory effect of *B. subtilis* 9407 against *A. citrulli* MH21.

**FIGURE 6:**
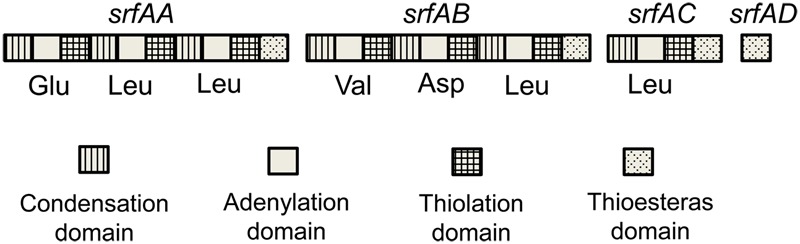
Domain organization of synthetase gene coding for surfactin in *B. subtilis* 9407. The amino acids recruited for the lipopeptide by each adenylation domain are listed under the domain organization.

**FIGURE 7:**
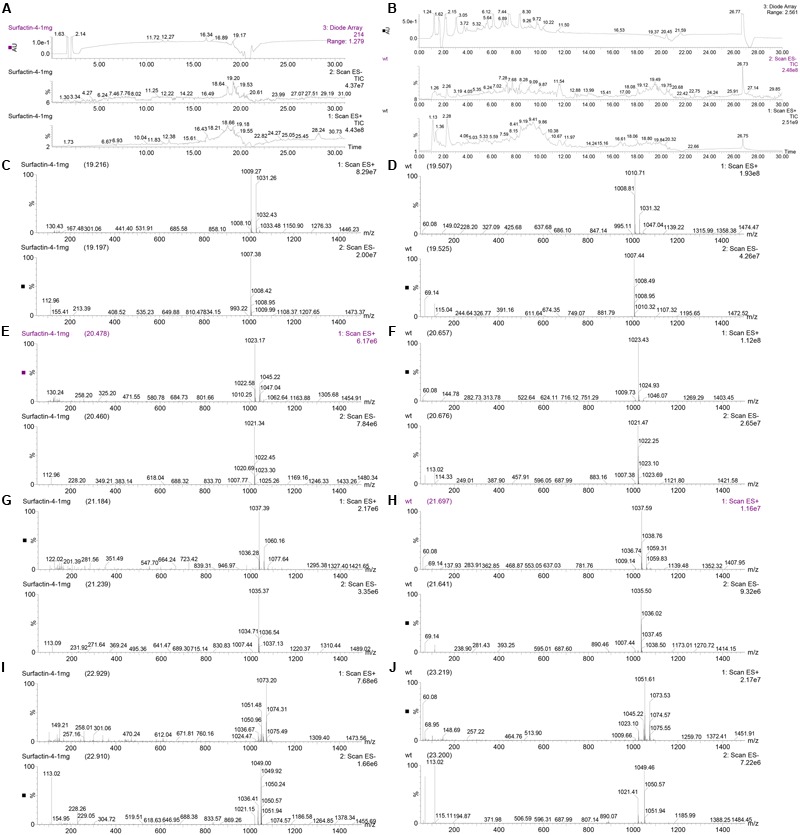
Analysis of lipopeptide crude extracts from *B. subtilis* 9407 and commercial standard for surfactin by LC-MS/MS. **(A)** The UV chromatogram, total ion flow diagram for negative ion mode (ES-) and positive ion mode (ES+) of commercial surfactin standard. **(B)** The UV chromatogram, total ion flow diagram for ES- and ES+ of lipopeptide crude extracts from *B. subtilis* 9407. **(C,E,G,I)** The mass spectrums of surfactin standard with ES- and ES+. **(D,F,H,J)** The mass spectrums of lipopeptide crude extracts from *B. subtilis* 9407 with ES- and ES+.

**FIGURE 8:**
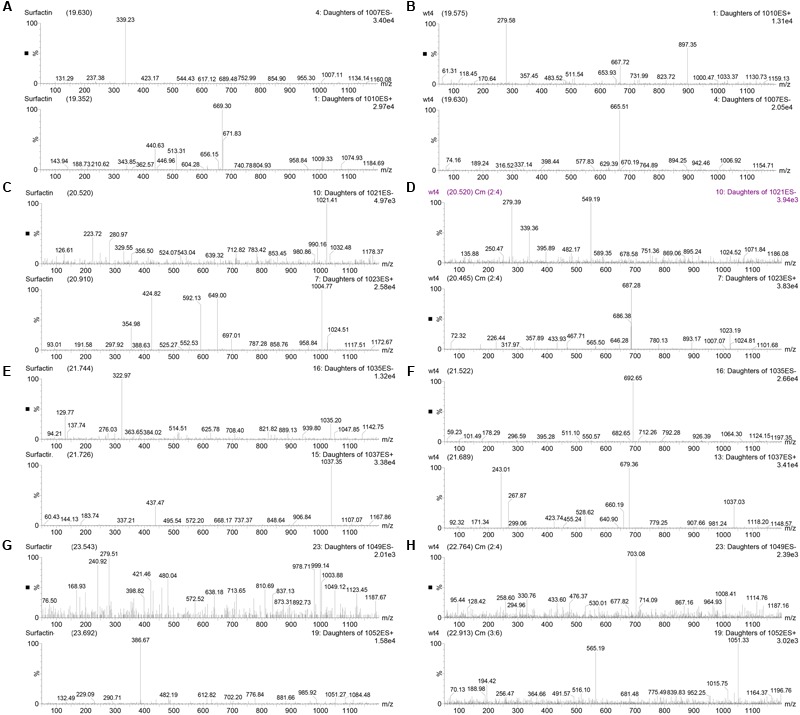
LC-MS/MS analysis of lipopeptide crude extracts from *B. subtilis* 9407 and commercial standard for surfactin. **(A,C,E,G)** The MS/MS spectrums of surfactin standard with negative ion mode (ES-) and positive ion mode (ES+). **(B,D,F,H)** The MS/MS spectrums of lipopeptide crude extracts from *B. subtilis* 9407 with ES- and ES+.

To further characterize surfactin produced by *B. subtilis* 9407, the complete surfactin gene cluster of *B. subtilis* 9407 was aligned with that in *B. subtilis* subsp. *subtilis* str. 168, *B. subtilis* subsp. *subtilis* 6051-HG and *B. subtilis* SG6, which were reported to produced surfactin A (Aleti et al., 2015, 2016). It was found that the complete surfactin gene cluster of *B. subtilis* 9407 displays 98.99% sequence identity to *B. subtilis* subsp. *subtilis* str. 168 and *B. subtilis* subsp. *subtilis* 6051-HG and 98.57% to *B. subtilis* SG6. AntiSMASH analysis of the surfactin cluster of *B. subtilis* 9407 predicted a lipopeptide sequence of _L_-Glu-_L_-Leu-_D_-Leu-_L_-Val-_L_-Asp-_D_-Leu-_L_-Ile, namely surfacrin A (**Figure 6**). Moreover, lipopeptide crude extracts from *B. subtilis* 9407 and commercial standard for surfactin, which is composed of a macrolide containing the heptapeptide Glu-Leu-Leu-Val-Asp-Leu-Leu (Lim et al., 2005; Aleti et al., 2016) were subjected to LC-MS/MS analysis. The LC-MS/MS analysis of *B. subtilis* 9407 lipopeptide crude extracts showed a series of four peaks with identical mass to surfactin standard (**Figure 7**). For each of the four compounds, the LC-MS/MS spectra with the same precursor m/z exhibited the quite similar fragmentation behavior was observed in the surfactin standard (**Figure 8**). These results indicated that the surfactin produced by *B. subtilis* 9407 is a mixture of C13- to C16-surfactin A.

**FIGURE 9:**
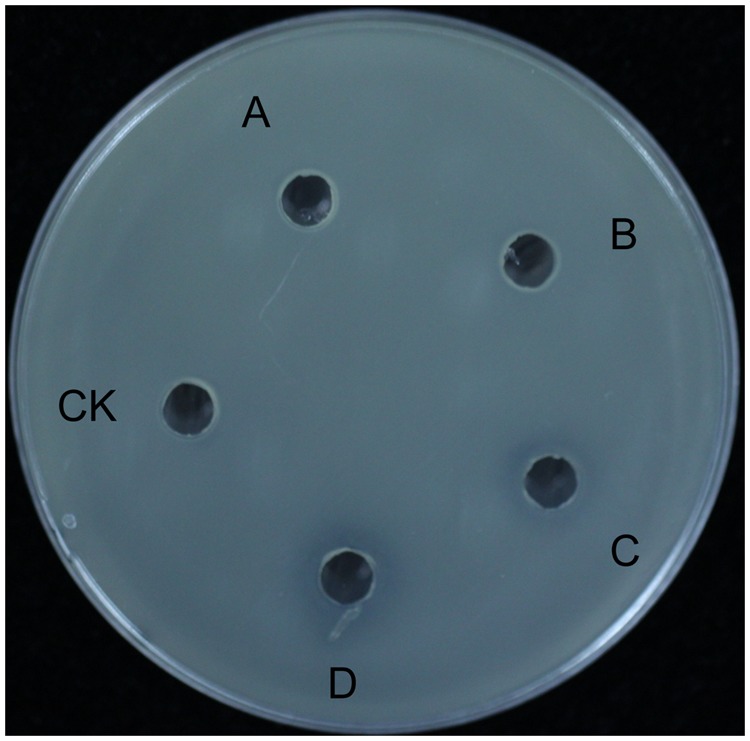
An antagonistic assay against *A. citrulli in vitro* by surfactin standard. **(CK)** methanol, **(A)** 1 μg surfactin, **(B)** 5 μg surfactin, **(C)** 25 μg surfactin and **(D)** 50 μg surfactin.

In order to determine the antibacterial activity of surfacrin, the MIC of surfactin standard was tested. It was found that the MIC of surfactin standard against *A. citrulli* MH21 was 100 μg/mL. Surfactin standard also showed antibacterial activity against *A. citrulli* MH21 on LB agar (**Figure 9**). These results suggested that surfactin standard has antibacterial activity against *A. citrulli* MH21.

### The Δ*srfAB* Mutant Showed a Defect in Biofilm Formation

**FIGURE 10:**
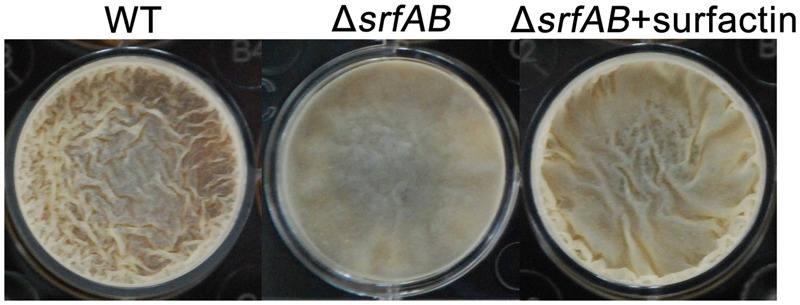
Biofilm formation of *B. subtilis* 9407(WT) and Δ*srfAB* with or without adding exogenous surfactin. WT and Δ*srfAB* were grown in MSgg medium for 72 h at 25°C in 12-well microtiter plates and images were taken. WT formed wrinkly floating pellicles at the liquid-air interface of liquid cultures in MSgg. Δ*srfAB* formed thinner pellicles. The biofilm formation of Δ*srfAB* was restored by adding 2 μL of the commercial surfactin (10 μg/mL).

Previous works have shown that surfactin triggers biofilm formation in *B. subtilis* (Romero, 2013; Zeriouh et al., 2014; Luo et al., 2015). We therefore asked whether this lipopeptide would also have a similar role in biofilm formation of *B. subtilis* 9407. We compared the biofilm formation phenotype of Δ*srfAB* and wild-type. The results showed that wild-type *B. subtilis* 9407 formed wrinkly floating pellicles at the liquid-air interface of liquid cultures in MSgg (**Figure 10**). In contrast, the Δ*srfAB* formed thinner pellicles in MSgg (**Figure 10**). Next, we tested whether the commercial surfactin could rescue the biofilm formation. We found that biofilm formation of Δ*srfAB* was restored in the presence of exogenously supplemented surfactin at 10 μg/mL (**Figure 10**). Therefore, our evidence suggested that surfactin production is important for biofilm formation in *B. subtilis* 9407.

### The Δ*srfAB* Mutant Lacked Swarming Motility

**FIGURE 11:**
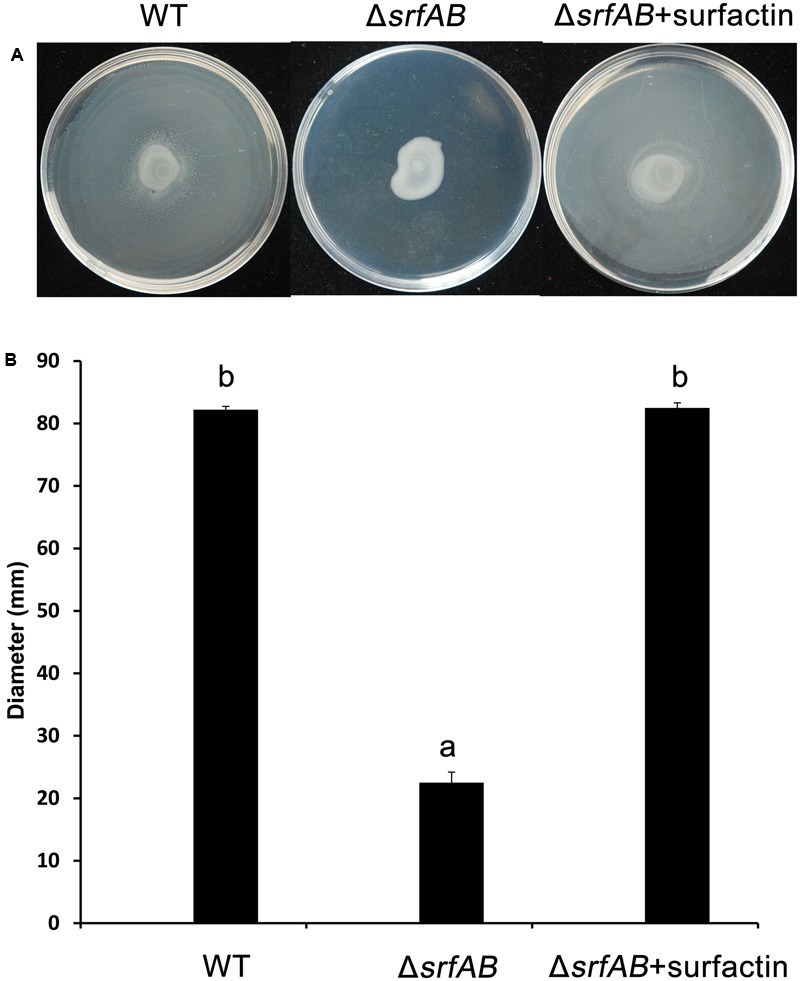
Swarming motility of *B. subtilis* 9407(WT) and Δ*srfAB* with or without adding exogenous surfactin as assessed in swarming agar plates. Δ*srfAB* showed a significant defect in swarming motility compared with WT. The swarming motility of Δ*srfAB* was restored by adding 2 μL of the commercial surfactin (10 μg/mL). **(A)** Swarming motility showed in swarming agar plates, **(B)** the diameter of swarming zone of *B. subtilis* 9407(WT) and Δ*srfAB* with or without adding exogenous surfactin.

Swarming motility is an important mechanism for bacterial colonization and a type of bacterial social movement related to surfactin production (Kearns and Losick, 2003). To analyze swarming motility, cell suspension was spotted at the center of the swarming agar plates, and the diameter of the swarming zone was measured. The wild-type strain showed excellent swarming motility and the plate almost fully covered by the swarming cells. While, the Δ*srfAB* showed a clear defect in swarming motility, with a 22.5 mm diameter of the swarming zone (**Figures 11A,B**). Based on the results of our external complementation experiments, where commercial surfactin rescued biofilm formation (**Figure 10**), we decided to analyze the effect of surfactin in the restoration of swarming motility by Δ*srfAB*. It was found that 10 μg/mL of commercial surfactin was necessary to restore swarming motility of Δ*srfAB* (**Figures 11A,B**). The above results indicated a significant role of the surfactin in swarming motility of *B. subtilis* 9407.

### Colonization Assay of Wild-type *B. subtilis* 9407 and the Δ*srfAB* Mutant on Melon Leaves and Roots

The implication of surfactin in the biofilm formation and swarming motility of *B. subtilis* suggested that this lipopeptide might have a role in the efficient colonization of melon leaves and roots. To test this hypothesis, the population dynamics of the wild-type and Δ*srfAB* on melon leaves and roots were evaluated over time. The results showed that wild-type and Δ*srfAB* could be isolated from the seedling tissues at 0, 5, 10, 15, and 20 days post inoculation, although the cell population of tested strains in the leaves and roots decreased continuously post inoculation (**Tables 3, 4**). The population of wild-type strain was higher in roots and leaves at each testing time point, where the highest level was reached at 5 days post inoculation, and the population was at a level of 3.56 × 10^4^ CFU/g (fresh weight) in the leaves (**Table 3**) and 7.44 × 10^4^ CFU/g (fresh weight) in the roots (**Table 4**). In contrast, the bacterial numbers of Δ*srfAB* were significantly lower than wild-type strain, and at 5 days post inoculation, the population was 1.67 × 10^4^ CFU/g (fresh weight) in the leaves (**Table 3**), 1.89 × 10^4^ CFU/g (fresh weight) in the roots (**Table 4**). Similar results were obtained at 10, 15, 20 days post inoculation (**Tables 3, 4**). In comparison with the wild type, Δ*srfAB* showed a two- to four-fold reduction and three- to ten-fold reduction in the number of leaves-colonizing cells and root-colonizing cells after inoculation, respectively. In general, the results strongly suggested that surfactin produced by *B. subtilis* 9407 affects the efficient colonization on melon leaves and roots.

**Table 3:** Colonization assay of wild-type *B. subtilis* 9407 and Δ*srfAB* mutant on melon leaves.

	Colonization ability				
Treatment	0 day (10^7^	5 days (10^4^	10 days (10^3^	15 days (10^3^	20 days (10^3^
	CFU/g leaves)	CFU/g leaves)	CFU/g leaves)	CFU/g leaves)	CFU/g leaves)
WT	7.93 ± 0.55^a^	3.56 ± 0.51^a^	4.82 ± 0.76^a^	3.74 ± 0.32^a^	2.66 ± 0.13^a^
Δ*srfAB*	7.70 ± 0.58^a^	1.67 ± 0.33^b^	1.27 ± 0.52^b^	0.96 ± 0.13^b^	0.78 ± 0.05^b^

*The colonization ability of wild-type *B. subtilis* 9407 and Δ*srfAB* mutant on melon leaves was determined 0–20 days post inoculation. Data are expressed as bacterial CFU per gram of leaves. The data are the average ± standard error from three replicates. Different letters represent a significant difference at *P* < 0.01.*

**Table 4:** Colonization assay of wild-type *B. subtilis* 9407 and Δ*srfAB* mutant on melon roots.

	Colonization ability				
Treatment	0 day (10^7^	5 days (10^4^	10 days (10^4^	15 days (10^4^	20 days (10^4^
	CFU/g roots)	CFU/g roots)	CFU/g roots)	CFU/g roots)	CFU/g roots)
WT	7.93 ± 0.55^a^	7.44 ± 0.38^a^	2.94 ± 0.47^a^	1.33 ± 0.07^a^	1.02 ± 0.05^a^
Δ*srfAB*	7.70 ± 0.58^a^	1.89 ± 0.19^b^	0.30 ± 0.06^b^	0.18 ± 0.03^b^	0.10 ± 0.01^b^

*The colonization ability of wild-type *B. subtilis* 9407 and Δ*srfAB* mutant on melon roots was determined 0-20 days post inoculation. Data are expressed as bacterial CFU per gram of roots. The data are the average ± standard error from three replicates. Different letters represent a significant difference at *P* < 0.01.*

### The Δ*srfAB* Mutant Was Defective in Its Biocontrol Activity

**FIGURE 12:**
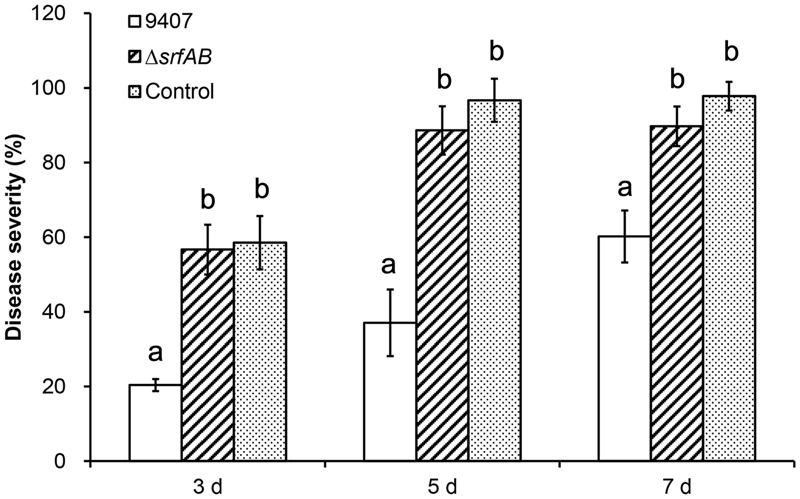
Biocontrol of *B. subtilis* 9407 and Δ*srfAB* against bacterial fruit blotch under greenhouse conditions. The germinated seeds were sowed in cell suspensions of *B. subtilis* 9407 and Δ*srfAB* at 10^7^ CFU/mL at room temperature for 30 min with gentle agitation. Seeds soaked in PBS buffer alone were used as the controls. After that the treated seeds were air-dried and sown in 600 mL black plastic pots (eight seeds per pot) filled with soil and vermiculite in a ratio of 2:1. The pots were placed in a greenhouse with the following conditions: 28–30°C, 60% humidity, and 16 h of light alternating with 8 h of darkness. After sown for 3 days, both sides of the leaves were sprayed with bacterial suspension of *A. citrulli* MH21(10^8^ CFU/mL). The seedlings were evaluated for BFB severity daily based on the disease index, as described previously. The disease index of each leaves was rated using a scale of 0–6, where 0, no symptom; 1, 10% or less necrotic lesions on leaves; 2–5, 11–25%; 26–50%; 51–75%, and 76–90% necrotic lesions on leaves, respectively; and 6, >90% necrosis of leaves. The disease severity and biocontrol efficacy were calculated as follows: Disease severity (%) = Σ the number of diseased leaves in each grade × grade/(total number of leaves investigated × the highest disease index) × 100. Biocontrol efficacy (%) = (incidence rate in the control - incidence rate in the *Bacillus*-treated group)/ incidence rate in the control × 100. Three pots were used for each replicate, and the values were recorded as the means of three replicates for each treatment. The experiments were repeated three times.

To examine whether surfactin is responsible for the biocontrol of *B. subtilis* 9407 against BFB *in vivo*, the wild-type *B. subtilis* 9407 and Δ*srfAB* were compared with the disease severity to evaluate the biocontrol activity. Three days after *A. citrulli* MH21 inoculation, the disease severity of melon seedlings that were pre-treated with PBS (control) was 58.52% (**Figure 12**). Treatment of melon seedlings with cells of the wild-type strain reduced the severity of BFB low to 20.37%. However, treatment with same amount of the Δ*srfAB* strain demonstrated a disease severity as high as 56.67%, showed no obvious differences compared with controls (**Figure 12**). Accordingly, Δ*srfAB* lost the capability of controlling BFB. Similar results were obtained 5 and 7 days post inoculation (**Figure 12**). These results demonstrated that surfactin produced by *B. subtilis* 9407 plays a major role in suppressing *A. citrulli*-induced BFB.

## Discussion

Biological control using microorganisms has been well known as a safe and efficient method for suppressing plant diseases (Lemos et al., 2016). In recent years, *Bacillus* spp., *A. avenae, P. anomala, Streptomyces* spp. and *B. amyloliquefaciens* 54 have been identified as biological control agents against BFB (Santos et al., 2006; Yaeram et al., 2006; Wang et al., 2009; Johnson et al., 2011; Jiang et al., 2015). However, the microorganisms on control of BFB is rare. To date, there are no reports about using *B. subtilis* as a biological control agent against BFB. Moreover, little is known about the biocontrol mechanism of microorganisms on control of BFB. In this study, *B. subtilis* 9407 demonstrated strong antibacterial activity against *A. citrulli* in the dual plate assay and 61.7% biocontrol efficacy on melon seedlings 4 days post inoculation under greenhouse conditions. Surfactin, which consists of C13- to C16-surfactin A, was the primary antibacterial compound of *B. subtilis* 9407, and it played a major role in biofilm formation, swarming motility, colonization and suppressing BFB. We propose that the biocontrol activity of *B. subtilis* 9407 is the results of the coordinated action of antibacterial activity and colonization. This study is the first report on the use of a *B. subtilis* strain as a potential biological control agent to control BFB and surfactin contributes to the biocontrol of BFB.

The mechanisms of biological control of *Bacillus* strains have often been associated with the production of different antimicrobial compounds (Ongena and Jacques, 2008). However, the mechanisms of biocontrol of BFB by *B. subtilis* are not clear. In the present study, *B. subtilis* 9407 inhibited the growth of *A. citrulli* MH21 *in vitro* (**Figures 1, 2**). The large inhibition zones could be due to the effects of antimicrobial compounds produced by *B. subtilis* 9407. These results suggested that the production of antibacterial compounds may be a mechanism of *B. subtilis* 9407 to suppress *A. citrulli.* To elucidate the mechanisms by which *B. subtilis* 9407 inhibits *A. citrulli* growth, we decided to mutate the selected candidate genes *srfAB* and *ppsB* responsible for the synthesis of surfactin and fengycin, respectively (Zeriouh et al., 2014; Fan et al., 2017). The Δ*srfAB* which was non-surfactin producer was almost completely defective in antibacterial activity against *A. citrulli* (**Figure 4**). These results suggested that surfactin may be a major antibacterial-active compound. Furthermore, the lipopeptide crude extracts from Δ*srfAB*, Δ*ppsB* and wild-type showed the similar results and demonstrated that surfactin was a primary active compound that led to the inhibitory effect of *B. subtilis* 9407 against *A. citrulli* (**Figure 5**). Surfactin has been isolated from members of the *Bacillus* genus, and it displays strong antimicrobial activity and inhibits the growth of a wide range of plant pathogens (Bais et al., 2004; Bacon et al., 2012). These results of this study demonstrated for the first time the antibacterial activity of surfactin against *A. citrulli*.

The surfactin family is comprised of three isoforms, which differ in their amino acid residue at position 7, namely surfactin A, B and C has a leucine, valine and isoleucine at position 7, respectively (Peypoux et al., 1999). Within each isoform, there are homologues differences in their branching and number of carbon atoms (Zhao et al., 2017). Different *Bacillus* strains produce varied surfactin. For example, surfactin C is the main component among *Bacillus subtilis* BC1212-produced surfactins (Hwang et al., 2007). *B. subtilis* EBS05 produces a mixture of the C12- to C15-surfactin A (Wen et al., 2011). Moreover, the quantity of surfactrin produced by different *Bacillus* strains is different. For example, Jia et al. (2015) reported that C12-, C13- and C16-surfactin are more abundant than C14- and C15-surfactin in *B. subtilis* B841. While, *B. subtilis* BS-37 usually produces C15-surfactin as the major component (Liu et al., 2015). Therefore, the quantity and isoforms of surfacrin are different in different members of the *Bacillus* genus. In this study, surfactin is a primary active compound of *B. subtilis* 9407 against *A. citrulli* MH21 *in vitro* (**Figures 4, 5**) and consists of C13- to C16-surfactin A (**Figures 7, 8**). At the same time, surfactin standard also showed antibacterial activity against *A. citrulli* MH21 (**Figure 9**). The difference in antibacterial activity of surfactin produced by *B. subtilis* 9407 and surfactin standard is required to determine. Further research is needed to determine the quantity and the exact chemical structure of the surfactin produced by *B. subtilis* 9407.

*B. subtilis* strains produce more than two dozen types of antimicrobial compounds to suppress the growth of phytopathogens (Stein, 2005). In our study, Δ*srfAB* also had an area of thinned growth of *A. citrulli* MH21 in dual culture assay (**Figures 4, 5**). Moreover, Δ*ppsB* showed a reduced antibacterial activity against *A. citrulli* MH21 (**Figures 4, 5**), indicating fengycin was one of antibacterial compounds. Additionally, we also found other peptide synthesis gene clusters in the genome of *B. subtilis* 9407 (data not shown). These findings indicate that besides fengycin and surfactin, other antimicrobial compounds may also play a role in antagonism against *A. citrulli*. Further research is required to determine other antimicrobial compounds.

Surfactin is also well known to involve in swarming motility and trigger biofilm formation. For example, a deficiency in surfactin production of *B. subtilis* UMAF6614 led to a reduction of swarming motility and biofilm formation (Zeriouh et al., 2014). Luo et al. (2015) reported that compared with wild type of *B. subtilis* 916, Δ*srf* deficient in surfactin production also showed substantially decreased in swarming motility and biofilm formation. In accordance with previous researches, we showed that Δ*srfAB* was deficient in swarming motility and biofilm formation (**Figures 10, 11**), which was restored in the presence of exogenously supplemented surfactin (**Figures 10, 11**), indicating that surfactin plays a major role in swarming motility and biofilm formation of *B. subtilis* 9407.

Successful colonization of biological control agents is considered a crucial step for successful biocontrol (Chowdhury et al., 2013; Weng et al., 2013). Many studies have illustrated that surfactin plays a vital role in colonization of *B. subtilis* on plant. For example, the mutant of *B. subtilis* 6051, which was unable to produce surfactin, failed to colonize on *Arabidopsis* roots (Bais et al., 2004). Moreover, Zeriouh et al. (2014) reported that *B. subtilis* UMAF6614 colonized on melon leaves due to the production of surfactin. Similarly, in our study, *B. subtilis* 9407 effectively colonized on melon leaves and roots, while Δ*srfAB* showed significantly decreased in the colonization on melon leaves and roots (**Tables 3, 4**). These results suggested that surfactin produced by *B. subtilis* 9407 affects the efficient colonization.

Previous studies have reported that surfactin plays essential roles in biological control of plant diseases (Alvarez et al., 2012; Deravel et al., 2014; Luo et al., 2015). However, the contribution of surfactin to the biocontrol of BFB *in vivo* is not clear. In this study, we performed biocontrol assays against BFB using wild type and Δ*srfAB*, and we determined that surfactin produced by *B. subtilis* 9407 played a major role in the biocontrol of BFB (**Figure 12**). Therefore, surfactin plays important roles in biocontrol of BFB via at least two mechanisms: as an antimicrobial agent and a stimulus for colonization. This result is consistent with previous study reporting that surfactin plays a primary role in biocontrol of tomato wilt disease by *B. subtilis* 3610 via at least two mechanisms describe above (Chen et al., 2013). The results of this study demonstrated for the first time the surfactin contributions to the biocontrol of BFB. So far, *Bacillus* spp., *A. avenae, Pichia anomala, Streptomyces* spp. are potential biological agents against BFB (Fessehaie and Walcott, 2005; Wang et al., 2009; Jiang et al., 2015) However, little is known about the biocontrol mechanism of them on control of BFB. Jiang et al. (2015) repoted that *B. amyloliquefaciens* 54 can significantly control the BFB by increasing the expression level of defense related gene PR1 and accumulation the hydrogen peroxide in the plant, when the watermelon were treated with *B. amyloliquefaciens* 54 at 1 × 10^8^ CFU/mL. In the present study, we propose that the biocontrol activity of *B. subtilis* 9407 is the results of the coordinated action of surfactin-mediated antibacterial activity and colonization, when this bacterium was used as a seed treatment at 1 × 10^7^ CFU/mL. The results of our study may provide a new biological control agent for controlling BFB and improve our understanding of the biocontrol mechanism of *B. subtilis* 9407.

## Conclusion

In conclusion, this study reported that *B. subtilis* 9407 efficiently controlled BFB that was caused by *A. citrulli in vitro* and *in vivo*. Moreover, the surfactin produced by *B. subtilis* 9407, which consisted of C13- to C16-surfactin A, was the primary antibacterial compound, and it played a major role in biofilm formation, swarming motility, colonization and biocontrol against BFB. We propose that the biocontrol activity of *B. subtilis* 9407 is the results of the coordinated action of surfactin-mediated antibacterial activity and colonization. This is the first report about the use of a *B. subtilis* strain as a potential biological control agent to control BFB through the production of surfactin.

## Author Contributions

HF carried out the main experiments, data analysis and wrote a manuscript draft. ZZ participated in the colonization assay and biocontrol analysis of BFB under greenhouse conditions. YL participated in experimental design and revised the manuscript. XZ participated in the construction of Δ*srfAB* mutant in *B. subtilis* 9407 and revised the manuscript. YD participated in the colonization assay. QW guided experimental design. All authors read and approved the final manuscript.

## Conflict of Interest Statement

The authors declare that the research was conducted in the absence of any commercial or financial relationships that could be construed as a potential conflict of interest. The reviewer JL declared a past co-authorship with several of the authors (YL and QW) to the handling Editor.
