# Supplementary material for: Biocontrol of Bacterial Fruit Blotch by Bacillus subtilis 9407 via Surfactin-Mediated Antibacterial Activity and Colonization
Source: Front Microbiol. 2017 Oct 11;8:1973. doi: 10.3389/fmicb.2017.01973 (PMC5641556; doi:10.3389/fmicb.2017.01973)
Supplement: Supplementary file 1 [file Data_Sheet_1.docx]

**Table S1**

Bacterial strains and plasmids used in this study.

| Strain and plasmid | Details | Reference or source |
| --- | --- | --- |
| ***B. subtilis*** |  |  |
| 9407 | Wild type, isolated from apple | (Fan et al., 2017) |
| ∆*srfAB* | *srfAB*::Tet^R^ | This study |
| ∆*ppsB* | *ppsB* unmarked deletion mutant of 9407 | (Fan et al., 2017) |
| 9407(pC-1) | 9407 with the plasmid pC-1, Cm^R^ | This study |
| ∆*srfAB*(pC-1) | ∆*srfAB* with the plasmid pC-1, Cm^R^ | This study |
| ***A. citrulli*** |  |  |
| MH21 | Wild type, isolated from melon, Amp^R^ | (Ren et al., 2014) |
| ***E. coli*** |  |  |
| DH5α | An *E. coli* host strain used for molecular cloning | Life Technologies |
| **Plasmids** |  |  |
| pMAD | Shuttle vector for allele replacement; containing the *bgaB* gene, which encodes a thermostable β-galactosidase; Amp^R^ and Ery^R^ | (Alvarez et al., 2012) |
| pGFP78 | Shuttle vector for *Bacillus* and *E. coli*; Amp^R^, Tet^R^; containing F78 promoter screened from *B. subtilis* ISW 1214 genomic DNA and GFP gene | (Gao et al., 2015) |
| pEBS | *Bacillus* suicide plasmid; Ery^R^ | (Wang et al., 2007) |
| pEBST | pEBS containing the Tet gene from pGFP78 | This study |
| pEBST-srfAB | 1,011-bp upstream and 1,213-bp downstream of *srfAB* gene PCR products amplified from *B. subtilis* 9407 using the primers srfAB-Up-F/srfAB-Up-R and srfAB-Dn-F/srfAB-Dn-R, respectively, cloned into the pEBST | This study |
| pMAD-srfAB | 4,173-bp Up-Tet-Dn fragment from pEBST-srfAB cloned into *Bgl*Ⅱ/*Mlu*Ⅰsite of pMAD | This study |
| pC-1 | Shuttle vector for *Bacillus* and *E. coli*, Amp^R^, Cm^R^; containing NCD promoter screened from *B. subtilis* NCD-2 genomic DNA and GFP gene | A gift of Prof. Ping Ma |

Tet^R^: Tetracycline resistance; Amp^R^: Ampicillin resistance; Cm^R^: chloramphenicol; Ery^R^: Erythromycin resistance.

**Table S2**

Oligonucleotides used in this study.

| Primer name | Primer sequence (5’-3’) (restriction sites underlined) | Reference or source |
| --- | --- | --- |
| Tet-F | AACTGCAGCTCTCTCCCAAAGTTGATCC | This study |
| Tet-R | GACTAGTGACACAGAAGAAGGCGATT | This study |
| srfAB-Up-F | ACGCGTCGACTCCGAAAGACCGCGAATCTG | This study |
| srfAB -Up-R | AACTGCAGAGCTGCTGATGCACAAATACCGTAC | This study |
| srfAB -Dn-F | GGACTAGTCGCTCAGCTCTTGATGGCAATC | This study |
| srfAB -Dn-R | CGAGCTCTACTTTGCTGACAATATGCGGGACA | This study |
| srfAB-F | GAAGATCTTCCGAAAGACCGCGAATCTG | This study |
| srfAB -R | CGACGCGTTACTTTGCTGACAATATGCGGGACA | This study |
| pC-1-F | TCTTTTTCTTGTAACTGGAACGGAGG | This study |
| pC-1-R | GCATGCCTGC AGGAGATTTA TTTGTA | This study |

**References**

Alvarez, F., Castro, M., Principe, A., Borioli, G., Fischer, S., Mori, G., and Jofre, E. (2012). The plant-associated *Bacillus amyloliquefaciens* strains MEP218 and ARP23 capable of producing the cyclic lipopeptides iturin or surfactin and fengycin are effective in biocontrol of sclerotinia stem rot disease. *J. Appl. Microbiol.* 112, 159-174. doi: 10.1111/j.1365-2672.2011.05182.x

Fan, H., Ru, J., Zhang, Y., Wang, Q., and Li, Y. (2017). Fengycin produced by *Bacillus subtilis* 9407 plays a major role in the biocontrol of apple ring rot disease. *Microbiol. Res.* 199, 89-97. doi: 10.1016/j.micres.2017.03.004

Gao, T., Foulston, L., Chai, Y., Wang, Q., and Losick, R. (2015). Alternative modes of biofilm formation by plant-associated *Bacillus cereus*. *Microbiologyopen* 4, 452-464. doi: 10.1002/mbo3.251

Ren, Z.G., Jiang, W.J., Ni, X.Y., Lin, M., Zhang, W., Tian, G.Z., and Zhang, L.Q. (2014). Multiplication of *Acidovorax citrulli* in planta during infection of melon seedlings requires the ability to synthesize leucine.  *Plant Pathol.* 63, 784-791. doi: 10.1111/ppa.12156

Wang, Y., Wang, H., Yang, C.-H., Wang, Q., and Mei, R. (2007). Two distinct manganese-containing superoxide dismutase genes in *Bacillus cereus*: their physiological characterizations and roles in surviving in wheat rhizosphere. *FEMS Microbiol. Lett.* 272, 206-213. doi: 10.1111/j.1574-6968.2007.00759.x
